# Relationship among Corneal Biomechanics, Anterior Segment Parameters, and Geometric Corneal Parameters

**DOI:** 10.1155/2016/8418613

**Published:** 2016-10-25

**Authors:** Sadık Görkem Çevik, Sertaç Argun Kıvanç, Berna Akova-Budak, Mediha Tok-Çevik

**Affiliations:** ^1^Department of Ophthalmology, Bursa Yuksek Ihtisas Training and Research Hospital, Bursa, Turkey; ^2^Department of Ophthalmology, School of Medicine, Uludag University, Bursa, Turkey; ^3^Department of Ophthalmology, Düzici State Hospital, Düzici, Turkey

## Abstract

*Purpose*. To investigate the relationship between corneal biomechanical parameters, anterior segment parameters, and geometric corneal parameters in a healthy Caucasian group.* Methods*. This retrospective study included the healthy eyes with best corrected visual acuity of at least 20/40 of 122 Caucasian subjects. The anterior segment parameters and geometric corneal parameters such as corneal volume, central corneal thickness, horizontal and vertical corneal radii, anterior and posterior steep, and flat keratometric values were measured with a Scheimpflug camera. The biomechanical properties were measured with Ocular Response Analyzer.* Results*. One hundred and twenty-two healthy Caucasian subjects (67 males, 55 females) with a mean age of 45.32 ± 20.23 were enrolled. Both corneal hysteresis and corneal resistance factor were positively correlated with CCT (*r* = 0.529, *p* < 0.001; *r* = 0.638, *p* < 0.001) and CV (*r* = 0.635, *p* < 0.001; *r* = 0.579, *p* < 0.001) and negatively correlated with age (*r* = −0.373, *p* < 0.001; *r* = −0.249, *p* < 0.001). Both in age-gender and multivariate models, CH and CRF had statistically significant negative association with the posterior steep *K* value.* Conclusions*. CH and CRF are negatively correlated with posterior steep and average posterior *K* values.

## 1. Introduction

Many factors affect corneal biomechanical properties in healthy eyes [1–3]. Anterior segment parameters such as central corneal thickness (CCT) are among these factors that have been intensively studied. Corneal hysteresis (CH) and corneal resistance factor (CRF) are strongly correlated with CCT [4–6]. A relation between refractive error and corneal biomechanical properties has also been reported [7]. The relationship between other anterior segment parameters such as corneal curvature (CC), corneal astigmatism (CA), corneal volume (CV), mean keratometric (*K*) value, and corneal biomechanical properties has been also investigated and the results are controversial [8–11]. Several studies in children reported that flatter corneal curvature was related to lower CH and CRF values [12, 13]. In this study, we aimed to investigate the relationship between corneal biomechanical parameters, anterior segment parameters, and geometric corneal parameters, including horizontal and vertical corneal radii and anterior and posterior steep and flat *K* values with 3 mm distance from the apex in a healthy Caucasian group.

## 2. Methods

### 2.1. Subjects

This retrospective study was carried out at Department of Ophthalmology of Bursa Yukses Ihtisas Training and Research Hospital. Initially, the medical records of 158 patients with best corrected visual acuity of at least 20/40 in one eye were reviewed. These patients were referred to the department for assessment of eligibility for refractive surgery, renewal of driving license, or obtaining medical report for any reason. The investigation was conducted in accordance with the Declaration of Helsinki and approved by institution. The exclusion criteria were history of previous ocular surgery and contact lens wearing, ocular inflammation, glaucoma, use of topical medication, and presence of diabetes mellitus and collagenous diseases. Of 158 patients, 122 healthy eyes of 122 subjects were included in the study.

### 2.2. Ocular Examinations

All the subjects underwent complete ophthalmologic examination including refraction and visual acuity measurement, biomicroscopic examination, intraocular pressure (IOP) measurement with a Goldmann applanation tonometer, and funduscopy.

### 2.3. Corneal Topography and Thickness Measurement

The anterior segment of the included eye of each subject was imaged with a 3D rotating Scheimpflug camera Sirius (CSO, Costruzione Strumenti Oftalmici, Florence, Italy) without application of any eyedrops. One of the authors performed all the measurements with Sirius. CCT, CV, anterior chamber depth (ACD), anterior chamber volume (ACV), horizontal and vertical corneal radii, CA, mean corneal *K*, and anterior and posterior corneal *K* values with 3 mm distance from the apex were measured.

### 2.4. Ocular Response Analyzer (ORA)

Four consecutive ORA (Reichert, Buffalo, New York, USA) measurements were performed and the averages of the measurements were taken. The high-quality readings defined by the manufacturer, because the force-in and force-out applanation signal peaks on the ORA waveform are fairly symmetrical in height, were accepted and recorded. The parameters used for analysis were CH, CRF, corneal compensated IOP (IOPcc), and Goldmann correlated IOP (IOPgc).

### 2.5. Statistical Analysis

The statistical analysis was performed by SPSS 22.0 statistical program. The relationship between variables was assessed with Pearson correlation analysis. Kolmogorov-Smirnov test was performed for the assessment of normal distribution of the data. Multivariate linear regression analysis was used for assessment of association of CH, CRF with anterior segment parameters, and geometric corneal parameters, including horizontal and vertical corneal radii, anterior and posterior steep and flat *K* values, IOP, and spherical equivalent.

## 3. Results

One hundred and twenty-two healthy Caucasian subjects (67 males, 55 females) with a mean age of 45.32 ± 20.23 were included in the study. The biomechanical properties, anterior segment parameters, and refraction values are given in Table 1.  Table 2 shows the relationship of CH, CRF, CCT, and CV with IOPgc, IOPcc, anterior segment parameters, and refraction values. Both CH and CRF were positively correlated with CCT (*r* = 0.529, *p* < 0.001; *r* = 0.638, *p* < 0.001) and CV (*r* = 0.635, *p* < 0.001; *r* = 0.579, *p* < 0.001) and negatively correlated with age (*r* = −0.373, *p* < 0.001; *r* = −0.249, *p* < 0.001). The corneal curvature and *K* values of both anterior and posterior surfaces are listed in Table 3. Correlations were found between CH and mean anterior *K* value, anterior flat *K* value, mean posterior *K* value, posterior steep *K* value, and vertical radius of CC. CRF was also correlated with mean posterior *K* value and posterior steep *K* value. No correlation was detected between CH, CRF, and mean *K* value (Table 4). In multivariate linear regression analysis, the associations between the corneal biomechanical parameters with *K* values of anterior and posterior surfaces and anterior segment parameters were investigated (Tables 5 and 6). Both in age-gender and multivariate models, CH and CRF had statistically significant negative association with the posterior step *K* value (Table 5). When IOP, ACD, ACV, CV, CCT, and SE were included in multivariate analysis, no association was detected between corneal biomechanical properties and *K* values. Age and IOP were negatively (*r* = −0.275, *p* = 0.016; *r* = −0.174, *p* = 0.048, resp.) and CCT was positively associated with CH (*r* = 0.477, *p* = 0.043). In addition, there were positive associations between IOP, ACD, and CCT with CRF (*r* = 0.323, *p* < 0.001; *r* = 0.350, *p* = 0.028; *r* = 0.741, *p* = 0.001, resp.) (Table 6).

## 4. Discussion

Previous studies showed that there is a correlation between corneal biomechanical properties and age. Though there are some controversial reports, most of the studies found a negative correlation between biomechanical properties and age [3, 10, 13–16].

In the present study, it is found that CH and CRF are negatively correlated with age in Caucasian population. Kotecha et al. also reported that CH and CRF are negatively correlated with age in a group of patients which included patients of different ethnic origin [10]. In a recent study, Jóhannesson et al. compared 50 Swedish young subjects with 43 elderly. They reported that CH was significantly lower in elderly group, but there was no difference in CRF between the groups [15]. Disaccordingly, in a study from Japan, no correlation was found between age and CH within 86 eyes of 43 healthy subjects with an age range of 19–64 years [3]. Ortiz et al. reported significant difference in CH between the youngest age group (9 to 14 years) and oldest age group (60 to 80 years) only [16].

Central corneal thickness is one of the important geometric corneal parameters that influence IOP measurement [17]. The biomechanical properties of cornea as CH and CRF are strongly correlated with CCT [4–6, 18]. The results of this study were in accordance with the literature. Besides CCT, the relationship of corneal curvature with corneal biomechanics was also studied. In children CC was found to be negatively correlated with CH and CRF [12, 13, 19]. In adults, the findings of the studies that investigated CC relation with corneal biomechanics are controversial. Franco and Lira found no relationship between CH, CRF, and CC in adults (20–63 years of age range) in 63 eyes [8]. Kamiya et al. reported similar result; they did not find any correlation between mean *K* value and CH and CRF in 86 eyes of 43 patients with a mean age of 39 years [3]. Wong and Lam found no correlation between mean *K* and CH and CRF in Chinese adults [9]. However, 2 different studies from Far East reported contradictory results. Narayanaswamy et al. found that flatter and thinner corneas were related to lower CH and CRF in 1136 Chinese patients (mean age 55 years) [2]. Hwang et al. reported results of 958 eyes of 958 patients with a mean age of 27 years. They concluded that CH and CRF were negatively correlated with mean radius of curvature [20]. Kotecha et al. found similar results in patients with different races with a mean age 50 years [10]. In other studies only CH was found to be associated with mean *K* [11, 21]. Diversity of the results in these studies may be related to the racial variety of the patients and age groups. Another reason for these results may be due to the different devices used for measurement for corneal parameters.

Apart from the other studies, this study investigated both steep and flat *K* values of anterior and posterior surface of the cornea. Although no correlation was found between mean *K* value and CH and CRF, it is noted that there are correlations with posterior steep *K* value and vertical radius of CC. CRF was also correlated with mean posterior *K* value and posterior steep *K* value. According to a multivariate regression model in the present study, posterior *K* values were found to be associated with CH and CRF. This finding may suggest that posterior surface of the cornea may affect corneal biomechanical properties more compared to anterior surface in normal eyes. In a recent study, it has been shown that CH and CRF were negatively associated with *K* max value in keratoconic eyes, but this correlation did not exist for normal eyes [22].

CV and its relation with corneal biomechanical properties have also been investigated in normal and keratoconic eyes in several studies [23, 24]. Hwang et al. found that corneal volume was positively correlated with CH, but not CRF [20]. Wong and Lam reported positive correlation both with CH and CRF [9]. In a recent study both CH and CRF were found to be correlated positively with CC and CV for the normal, keratoconic, and crosslinked eyes [22]. In this study, there are also positive correlations noted between CH, CRF, and CV.

In conclusion, CH and CRF are positively correlated with CCT and CV and negatively correlated with age in a healthy Caucasian population. It is also shown with this study that CH and CRF are negatively correlated with posterior steep and average posterior *K* values. Anterior and posterior *K* values account for 16.6% and 9.5% of variation in CH and CRF, respectively.

## Figures and Tables

**Table 1:** The biomechanical properties, anterior segment parameters, and refraction values of healthy subjects.

	Mean	Standard deviation
Age (year)	45.32	20.23
CH (mmHg)	10.67	1.86
CRF (mmHg)	10.89	1.79
IOPg (mmHg)	16.35	4.08
IOPc (mmHg)	16.33	4.26
CCT (microns)	552.19	37.21
CV (mm^3^)	60.69	4.42
ACD (mm)	2.79	0.52
ACV (mm^3^)	161.06	47.19
Spherical value (D)	−0.34	2.45
Cylindrical value (D)	−0.75	0.56
SE (D)	−.54	2.15

CH: corneal hysteresis; CRF: corneal resistance factor; IOPgc: intraocular pressure (Goldman correlated); IOPcc: intraocular pressure (cornea compensated); CCT: central corneal thickness; CV: corneal volume; ACD: anterior chamber depth; ACV: anterior chamber volume; SE: spherical equivalent, D: diopter.

**Table 2:** The relationship of CH, CRF, CCT, and CV with IOPgc, IOPcc, anterior segment parameters, and refraction values.

	Age		CH		CRF		CCT		CV	
	*r* value	*p* value	*r* value	*p* value	*r* value	*p* value	*r* value	*p* value	*r* value	*p* value
CH	−0.373	<0.001	N/A	N/A	0.752	<0.001	0.529	<0.001	0.635	<0.001
CRF	−0.249	0.015	0.752	<0.001	N/A	N/A	0.638	<0.001	0.579	<0.001
IOPgc	0.137	0.185	−0.202	0.046	0.506	<0.001	0.247	0.014	0.041	0.692
IOPcc	0.279	0.005	−0.620	<0.001	0.066	0.517	−0.040	0.697	−0.255	0.011
CCT	−0.263	0.01	0.529	<0.001	0.638	<0.001	N/A	N/A	0.865	<0.001
CV	−0.349	0.001	0.635	<0.001	0.579	<0.001	0.865	<0.001	N/A	N/A
ACD	−0.454	<0.001	0.180	0.076	0.123	0.227	−0.103	0.311	0.009	0.927
ACV	−0.565	<0.001	0.158	0.121	0.087	0.393	0.024	0.813	0.073	0.475
IOP	−0.14	0.915	0.004	0.966	0.506	<0.001	0.295	0.003	0.182	0.072
Spheric value	0.262	0.008	−0.148	0.147	−0.121	0.237	−0.172	0.091	−0.163	0.110
Cylindrical value	−0.278	0.007	0.119	0.244	0.193	0.056	0.256	0.011	0.250	0.013
SE	0.231	0.025	−0.135	0.186	−0.097	0.341	−0.128	0.204	−0.137	0.174

CH: corneal hysteresis; CRF: corneal resistance factor; IOPgc: intraocular pressure (Goldman correlated); IOPcc: intraocular pressure (cornea compensated); CCT: central corneal thickness; CV: corneal volume; ACD: anterior chamber depth; ACV: anterior chamber volume; SE: spherical equivalent; IOP: intraocular pressure.

**Table 3:** The corneal curvature and keratometric values of both anterior and posterior surfaces.

	Mean	Standard deviation
Horizontal radius of CC (mm)	7.86	.27
Vertical radius of CC (mm)	7.73	.27
Average radius of CC (mm)	7.79	.26
Anterior flat *K* value (D)	42.92	1.44
Anterior steep *K* value (D)	43.84	1.53
Average anterior *K* value (D)	43.39	1.43
Posterior flat *K* value (D)	−5.96	1.26
Posterior steep *K* value (D)	−6.41	.30
Average posterior *K* value (D)	−6.19	.66
Average flat *K* value (D)	41.67	1.34
Average steep *K* value (D)	42.50	1.48
Average *K* value (D)	42.09	1.37
Corneal astigmatism (D)	0.86	0.66

*K*: keratometric; CC: corneal curvature.

**Table 4:** The relationship of corneal biomechanical properties, age, CCT, and CV with keratometric values of anterior and posterior corneal surface.

		Age	CH	CRF	CCT	CV
Horizontal radius of CC	*r*	−0.247	−0.181	−0.101	0.096	−0.189
	*p*	0.012	0.085	0.362	0.351	0.065
Vertical radius of CC	*r*	−0.165	−0.217	−0.072	0.052	−0.196
	*p*	0.123	0.033	0.454	0.589	0.057
Average radius of CC	*r*	−0.211	−0.201	−0.093	0.077	−0.199
	*p*	0.034	0.043	0.387	0.445	0.047
Anterior flat *K* value	*r*	0.213	0.206	0.108	−0.069	0.222
	*p*	0.032	0.046	0.311	0.532	0.027
Anterior steep *K* value	*r*	0.198	0.197	0.074	−0.076	0.175
	*p*	0.055	0.059	0.503	0.455	0.095
Average anterior *K* value	*r*	0.209	0.206	0.095	−0.073	0.204
	*p*	0.032	0.044	0.375	0.472	0.043
Posterior flat *K* value	*r*	−0.125	0.182	0.128	0.157	0.078
	*p*	0.240	0.091	0.208	0.119	0.443
Posterior steep *K* value	*r*	−0.103	−0.362	−0.254	−0.143	−0.478
	*p*	0.287	<0.001	0.013	0.152	<0.001
Average posterior *K* value	*r*	−0.143	0.085	0.065	0.123	−0.034
	*p*	0.175	0.419	0.528	0.254	0.737
Average flat *K* value	*r*	0.160	0.184	0.069	−0.095	0.183
	*p*	0.103	0.085	0.497	0.342	0.069
Average steep *K* value	*r*	0.243	0.156	0.047	−0.151	0.080
	*p*	0.019	0.125	0.642	0.138	0.489
Average *K* value	*r*	0.212	0.175	0.060	−0.127	0.129
	*p*	0.034	0.088	0.560	0.207	0.201
Corneal astigmatism	*r*	0.192	−0.048	−0.092	−0.165	−0.203
	*p*	0.061	0.702	0.419	0.108	0.041

*K*: keratometric; CC: corneal curvature.

**Table 5:** Multivariate linear regression analysis of corneal biomechanical properties and keratometric values of anterior and posterior corneal surfaces.

	CH				CRF			
	Age-gender model		Multivariate model		Age-gender model		Multivariate model	
	Standardized (ß)/ unstandardized (B) Coefficient (SE)	*p* value/95% CI	Standardized (ß)/ unstandardized (B) Coefficient (SE)	*p* value/95% CI	Standardized (ß)/ unstandardized (B) Coefficient (SE)	*p* value	Standardized (ß)/ unstandardized (B) Coefficient (SE)	*p* value
Adjusted *r*	0.329		0.196		0.157		0.123	
Gender	−0.007/−0.024 (0.294)	0.934/−0.607–0.558	—	—	−0.030/−0.107 (0.320)	0.739/−0.740–0.526	—	—
Age	−0.383/−0.035 (0.007)	<0.001/−0.049–−0.021	—	—	−0.219/−0.019 (0.008)	0.013/−0.034–−0.004	—	—
AFKV	0.099/0.130 (0.205)	0.527/−0.276–0.536	0.021/0.027 (0.223)	0.903/−0.414–0.469	0.103/0.131 (0.223)	0.559/−0.311–0.572	0.055/0.071 (0.226)	0.753/−0.376–0.518
ASKV	−0.243/−0.305 (0.213)	0.161/−0.721–0.121	−0.302/−0.373 (0.232)	0.201/−0.833–0.087	−0.399/−0.478 (0.231)	0.068/−0.935–0.020	−0.435/−0.520 (0.235)	0.065/−0.985–−0.055
PFKV	0.137/0.220 (0.126)	0.082/−0.029–0.469	0.154/0.247 (0.137)	0.046/−0.025–0.518	0.085/0.131 (0.136)	0.337/−0.0.139–0.402	0.092/0.143 (0.138)	0.303/−0.131–0.418
PSKV	−0.495/−3.545 (0.859)	<0.001/−5.247–−1.843	−0.653/−4.16 (0.924)	<0.001/−5.934–−2.304	−0.560/−3.463 (0.933)	<0.001/−5.312–−1.614	−0.611/−3.779 (0.935)	<0.001/−5.631–−1.926

CH: corneal hysteresis; CRF: corneal resistance factor, SE: standard error, CI: confidence interval, AFKV: anterior flat keratometric value, ASKV: anterior steep keratometric value, PFKV: posterior flat keratometric value, and PSKV: posterior steep keratometric value.

**Table 6:** Multivariate regression analysis of CH, CRF and age, IOP, anterior segment parameters, and keratometric values of anterior and posterior corneal surfaces.

	CH						CRF					
	Age-gender model		Corneal keratometric value model		Multivariate model		Age-gender model		Corneal keratometric value model		Multivariate model	
	Standardized (ß)/ unstandardized (B) Coefficient (SE)	*p* value	Standardized (ß)/ unstandardized (B) Coefficient (SE)	*p* value	Standardized (ß)/ unstandardized (B) Coefficient (SE)	*p* value/95% CI	Standardized (ß)/ unstandardized (B) Coefficient (SE)	*p* value	Standardized (ß)/ unstandardized (B) *p* value Coefficient (SE)	*p* value	Standardized (ß)/ unstandardized (B) Coefficient (SE)	*p* value
Adjusted *r*	0.455		0.432		0.416		0.545		0.544		0.537	
Gender	0.086/0.297 (0.304)	0.357/−0.343–0.945	—	—	0.114/0.375 (0.316)	0.235/−0.256–1.115	0.020/0.065 (0.279)	0.836/−0.481–0.621	—	—	0.033/0.129 (0.282)	0.649/−0.426–0.678
Age	−0.275/−0.034 (0.013)	0.016/−0.044–−0.005	—	—	−0.217/−0.017 (0.008)	0.036/−0.038–−0.002	−0.124/−0.011 (0.009)	0.256/−0.031–0.007	—	—	−0.058/−0.005 (0.008)	0.546/−0.027–0.019
IOP	−0.174/−0.094 (0.049)	0.048/−0.202–−0.001	−0.142/−0.079 (0.048)	0.135/−0.168–0.027	−0.172/−0.08 (0.047)	0.061/−0.19–0.005	0.323/0.173 (0.04)	<0.001/0.093–0.262	0.346/0.181 (0.045)	<0.001/0.11–0.27	0.356/0.190 (0.041)	<0.001/0.125–0.277
ACD	0.324/1.067 (0.563)	0.055/−0.045–2.135	0.366/1.267 (0.577)	0.043/0.025–2.334	0.416/1.338 (0.551)	0.02/0.248–2.468	0.350/1.102 (0.492)	0.028/0.109–2.082	0.375/1.139 (0.49)	0.017/0.172–2.221	0.421/1.322 (0.465)	0.006/0.381–2.274
ACV	−0.129/−0.005 (0.006)	0.393/−0.017–0.007	−0.146/−0.006 (0.005)	0.464/−0.025–0.012	−0.353/−0.015 (0.007)	0.063/−0.03–0.002	−0.171/−0.006 (0.006)	0.413/−0.020–0.007	−0.119/−0.005 (0.006)	0.521/−0.019–0.009	−0.267/−0.01 (0.005)	0.126/−0.024–0.003
CV	0.035/0.017 (0.111)	0.915/−0.211–0.242	0.372/0.145 (0.1)	0.159/−0.067–0.356	0.437/0.166 (0.066)	0.009/0.042–0.332	−0.259/−0.099 (0.096)	0.298/−0.297–0.091	−0.116/−0.047 (0.09)	0.645/−0.226–0.128	0.124/0.046 (0.06)	0.474/−0.077–0.167
CCT	0.477/0.025 (0.009)	0.043/0.011–0.057	0.264/0.012 (0.11)	0.257/−0.013–0.034	0.216/0.010 (0.008)	0.278/−0.012–0.038	0.741/0.036 (0.01)	0.001/0.016–0.055	0.668/0.035 (0.008)	0.002/0.017–0.058	0.481/0.022 (0.006)	0.003/0.009–0.042
SE	−0.014/−0.008 (0.072)	0.912/−0.157–0.142	0.019/0.015 (0.078)	0.903/−0.145–0.172	−0.062/−0.038 (0.072)	0.596/−0.192–0.16	0.112/0.083 (0.062)	0.235/−0.051–0.206	0.119/0.092 (0.065)	0.183/−0.056–0.235	0.071/0.005 (0.061)	0.431/−0.077–0.177
AFKV	0.064/0.081 (0.170)	0.634/−0.256–0.417	−0.074/−0.078 (0.215)	0.743/−0.487–0.327	—	—	0.082/0.101 (0.19)	0.624/−0.278–0.477	0.032/0.035 (0.18)	0.867/−0.332–0.410	—	—
ASKV	−0.049/−0.058 (0.190)	0.759/−0.435–0.318	0.174/0.245 (0.230)	0.395/−0.265–0.675	—	—	−0.052/−0.059 (0.212)	0.814/−0.472–0.350	0.023/0.021 (0.195)	0.894/−0.374–0.399	—	—
PFKV	0.055/0.086 (0.096)	0.374/−0.105–0.276	0.132/0.194 (0.121)	0.137/−0.057–0.510	—	—	0.076/0.103 (0.101)	0.336/−0.106–0.308	0.085/0.113 (0.100)	0.267/−0.084–0.312	—	—
PSKV	−0.319/−1.971 (0.947)	0.126/−3.849–−0.093	−0.093/−0.539 (1.177)	0.648/−2.906–1.792	—	—	−0.245/−1.426 (1.071)	0.205/−3.539–0.715	−0.178/−1.001 (1.005)	0.323/−3.001–0.996	—	—

CH: corneal hysteresis; CRF: corneal resistance factor; CCT: central corneal thickness; CV: corneal volume; ACD: anterior chamber depth; ACV: anterior chamber volume; SE: spherical equivalent; IOP: intraocular pressure.
